# Mitochondrial pseudogenes in the nuclear genome of *Aedes aegypti *mosquitoes: implications for past and future population genetic studies

**DOI:** 10.1186/1471-2156-10-11

**Published:** 2009-03-06

**Authors:** Thaung Hlaing, Willoughby Tun-Lin, Pradya Somboon, Duong Socheat, To Setha, Sein Min, Moh Seng Chang, Catherine Walton

**Affiliations:** 1 Faculty of Life Sciences, University of Manchester, Oxford Road, Manchester M13 9PT, UK; 2 Medical Entomology Research Division, Department of Medical Research (Lower Myanmar), 5 Ziwaka Road, Dagon P.O., Yangon 11191, Myanmar; 3 Department of Parasitology, Faculty of Medicine, Chiang Mai University, Chiang Mai 50200, Thailand; 4 National Centre for Malaria, Parasitology and Entomology, Phnom Penh, Cambodia; 5 WHO – Western Pacific Regional Office, Phnom Penh, Cambodia

## Abstract

**Background:**

Mitochondrial DNA (mtDNA) is widely used in population genetic and phylogenetic studies in animals. However, such studies can generate misleading results if the species concerned contain nuclear copies of mtDNA (Numts) as these may amplify in addition to, or even instead of, the authentic target mtDNA. The aim of this study was to determine if Numts are present in *Aedes aegypti *mosquitoes, to characterise any Numts detected, and to assess the utility of using mtDNA for population genetics studies in this species.

**Results:**

BLAST searches revealed large numbers of Numts in the *Ae. aegypti *nuclear genome on 146 supercontigs. Although the majority are short (80% < 300 bp), some Numts are almost full length mtDNA copies. These long Numts are not due to misassembly of the nuclear genome sequence as the Numt-nuclear genome junctions could be recovered by amplification and sequencing. Numt evolution appears to be a complex process in *Ae. aegypti *with ongoing genomic integration, fragmentation and mutation and the secondary movement of Numts within the nuclear genome.

The PCR amplification of the putative mtDNA nicotinamide adenine dinucleotide dehydrogenase subunit 4 (*ND4*) gene from 166 Southeast Asian *Ae. aegypti *mosquitoes generated a network with two highly divergent lineages (clade 1 and clade 2). Approximately 15% of the *ND4 *sequences were a composite of those from each clade indicating Numt amplification in addition to, or instead of, mtDNA. Clade 1 was shown to be composed at least partially of Numts by the removal of clade 1-specific bases from composite sequences following enrichment of the mtDNA. It is possible that all the clade 1 sequences in the network were Numts since the clade 2 sequences correspond to the known mitochondrial genome sequence and since all the individuals that produced clade 1 sequences were also found to contain clade 2 mtDNA-like sequences using clade 2-specific primers. However, either or both sets of clade sequences could have Numts since the BLAST searches revealed two long Numts that match clade 2 and one long Numt that matches clade 1. The substantial numbers of mutations in cloned *ND4 *PCR products also suggest there are both recently-derived clade 1 and clade 2 Numt sequences.

**Conclusion:**

We conclude that Numts are prevalent in *Ae. aegypti *and that it is difficult to distinguish mtDNA sequences due to the presence of recently formed Numts. Given this, future population genetic or phylogenetic studies in *Ae. aegypti *should use nuclear, rather than mtDNA, markers.

## Background

Mitochondrial DNA (mtDNA) has been used extensively over the last three decades in population genetic and phylogenetic studies in a wide range of animals from *Drosophila *to humans (e.g. [1-4]). The advantages of mtDNA include its general lack of recombination that results in a single demographic history for the whole molecule, and high copy number which allows ease of amplification [5]. Further, the relatively high mutation rate of mtDNA generates correspondingly high levels of polymorphism and divergence [5]. This makes mtDNA particularly informative for the determination of genetic population structure and inference of population history within species as well as for deducing phylogenetic relationships between closely related species. MtDNA has been applied to many insect taxa including bees [6] and ants [7], in addition to medically important insects such as *Anopheles *[8-10] and *Aedes *mosquitoes [11,12] where it is particularly important to estimate gene flow for vector control purposes [13,14]. Most recently, the use of mtDNA sequences has been proposed for several DNA bar-coding initiatives for taxonomic identification and biodiversity assessment [15].

However, mtDNA also has its disadvantages. MtDNA data can be misleading in population and phylogenetic studies as it is particularly prone to selective sweeps [16]; it can introgress with relative ease between species [17]; and its population dynamics may be driven by intracellular symbionts [18]. One problem that can be particularly difficult to detect is the presence of nuclear mitochondrial pseudogenes (Numts) [19,20]. Numts result from the translocation of mitochondrial sequences from the mitochondrial genome into the nuclear genome and, once integrated, these non-functional sequences accumulate mutations freely. The potential for Numt amplification in addition to, or even instead of, the authentic target mtDNA sequence can seriously confound population genetic and phylogenetic analyses (reviewed in [21,22]). For example, the mistaken inclusion of Numt sequences in an mtDNA phylogeny resulted in incorrect phylogenetic relationships being proposed within the South American bird genus *Scytalopus *[23,24]. The high prevalence of Numts in gorillas has also been problematic as they initially obscured the presence of two genetically divergent groups of gorillas which has important implications for understanding their evolutionary history and future conservation [25-27].

In 2001, over 82 different eukaryotes, including 20 insect species, were known to have Numts [22]. However, there have been numerous reports of Numts since then, for example, in gorillas [25], domestic cats [28], chickens [29], and several insects such as bees [30] and ants [31]. Although taxonomically widespread, there is substantial variation among species in Numt copy number [32]. Even within insects, Numt copy number varies greatly with high numbers in *Tribolium *flour beetles, honeybee and the brown mountain grasshopper [30,33], but few or none in *Drosophila *and *Anopheles *mosquitoes [32]. Although a positive correlation between Numt copy number and genome size is not clear cut [29,32], genome size may partially explain taxonomic variation in Numt copy number [22]. In this context it worth noting that the genome of *Ae. aegypti *is particularly large, 1.3 Gb [34]. Numt fragments tend to be small, with the vast majority (>90%) less than 1 kb, and often less than 100 bp [30,32,35]. The longest Numts have been found in species with high Numt copy number, such as humans, mouse and rice [32]. Controlling for size of mtDNA, the largest reported Numt to date is 14,654 bp in humans which corresponds to ~88% of the mitochondrial genome [36].

MtDNA has been widely used to study genetic population structure in *Ae. aegypti *mosquitoes, the organism of study here [37-41]. This mosquito is thought to have spread relatively recently from its ancestral home in Africa to the rest of the Tropics where they are important vectors of dengue and yellow fever [42,43]. An understanding of genetic population structure and gene flow in *Ae. aegypti *is therefore of particular importance as this information is required for vector control [44-46]. The presence of Numts in *Ae. aegypti *could therefore seriously confound the interpretation of previous population genetic studies in *Ae. aegypti *as well as restrict the use of mtDNA in future studies.

Numt presence can manifest itself in several ways including: PCR ghost bands; extra bands in restriction profiles; sequence ambiguities; and unexpected phylogenetic placement ([22] and references therein). In our mitochondrial sequences of this species we found a significant proportion of sequence ambiguities suggestive of the presence of Numts. In general, previous authors have not reported sequence ambiguities in *Ae. aegypti*. The study by Paduan and Ribolla [47] is one exception but here the sequence ambiguities were attributed to heteroplasmy (where wild type and mutant mitochondrial genomes co-exist in a cell [48]). Another possible indication of the presence of Numts is the deep clade structure within many worldwide populations of *Ae. aegypti*. Two divergent mtDNA clades have been reported from: Mexico [37,49]; Thailand [38]; Venezuela [41]; Australia [50]; and the Americas [40]. The aims of this study are therefore to determine if Numts are present in *Ae. aegypti *mosquitoes, to characterise any Numts present, and to assess the utility of using mtDNA for population genetics studies in this species. Using a combined experimental and bioinformatics approach utilizing the recent genome sequence of *Ae. aegypti *(available at ) [34] we show here that Numts are prevalent in *Ae. aegypti *and that many previous studies have likely included Numt sequences in their mtDNA sequences. Finally, we discuss the implications of the use of mtDNA in future population genetic studies of this species.

## Results

### Numt identification using BLAST searches

**Figure 1:**
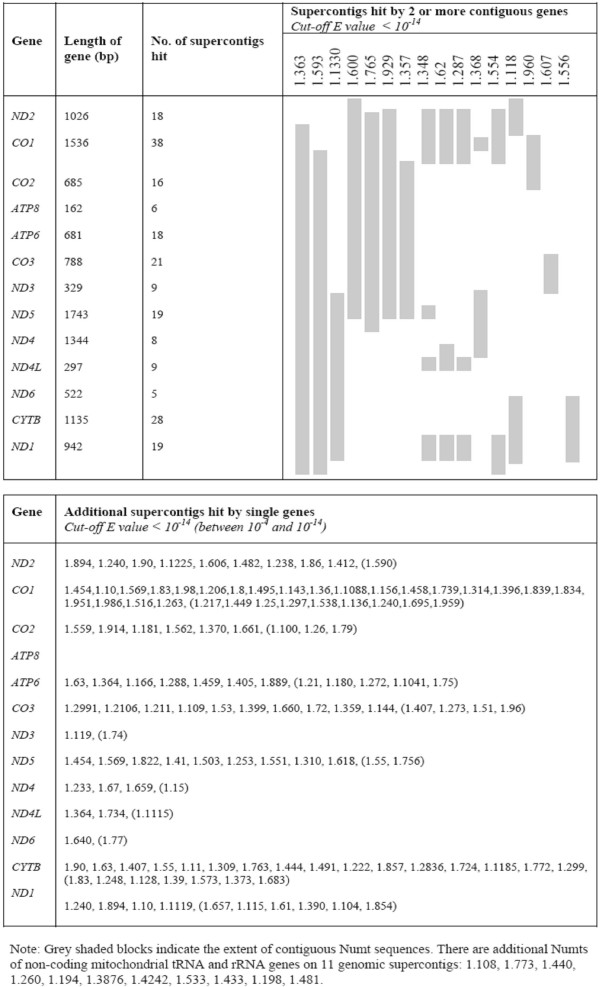
**Numbers and lengths of Numts of 13 protein-coding mitochondrial genes detected by BLAST searches of the *Ae. aegypti *genome sequence**.

Nucleotide BLAST searches of the *Ae. aegypti *genome sequence with each of the protein-coding genes from the mtDNA sequence of *Ae. aegypti *(GenBank accession number: EU352212) identified Numts on 135 different genomic supercontigs (using a cutoff *E *value of 10^-4^) or 98 supercontigs (using a cutoff *E *value of 10^-14^) (Figure 1). An additional 11 supercontigs were found to contain Numts derived from the tRNA and rRNA genes. Any overlap of supercontigs due to misassembly would result in fewer, but longer, Numts being detected. Assuming that overlap of the supercontig sequences is minimal, the number and size of Numts detected here should be reliable estimates. The Numts appeared to have originated from all over the mitochondrial genome, with longer genes typically having given rise to more Numts. Sequence identities of Numts with the mtDNA sequence ranged from 83% to 100%. The shortest Numts found were 28 bp for protein coding regions (e.g. a *CO1 *fragment on supercontig 1.695, *E *value = 3 × 10^-5^) and 63 bp for non-protein coding regions (tRNA-Arg and tRNA-Ala on supercontig 1.774, *E *value = 4 × 10^-25^). The majority of Numts were less than 300 bp in length (Table 1).

**Table 1:** Size distribution of *Ae. aegypti *Numts (protein coding, tRNA and rRNA) detected by BLAST searches

Size (bp)	Number of Numts
21–40	23
41–80	56
81–160	38
161–320	23
321–640	7
641–1280	5
1281–2560	8
2561–5120	2
5121–10240	4
13,086	1
15,455	1

Although the majority of Numts are small in length, the penultimate column in Figure 1 shows that 16 supercontigs contain Numts that span two or more genes. Of these, 7 supercontigs have sequences corresponding to 6 or more contiguous mtDNA genes. The remaining supercontigs contain one to three smaller fragments of mtDNA-like sequence with each fragment spanning no more than three genes. For these longer Numts there appears to be some pattern across supercontigs in the genetic make up of the Numts. For example, supercontigs 1.348, 1.62 and 1.287 all contain a long Numt that spans the contiguous genes *ND1*, *ND2*, and *CO1 *as well as part of the non-contiguous *ND4L *gene. In addition, supercontigs 1.600, 1.765 and 1.929 span the contiguous genes *ND2*, *CO1*, *CO2*, *ATP8*, *ATP6*, *CO3*, *ND3 *and *ND5*. With the exception of the first two, this set of contiguous genes is also contained by the Numt on supercontig 1.357. Although these contiguous genes were detected by BLAST using each protein coding gene individually, alignments between the mtDNA and the supercontigs showed that in all cases the Numts were contiguous across all coding and non-coding regions with *E *values < 10^-14^. Despite their apparent similarity, the start and end positions of these long Numts differ in every case. There are also two supercontigs (1.363 and 1.593) that contain almost full length mtDNA copies.

**Figure 2:**
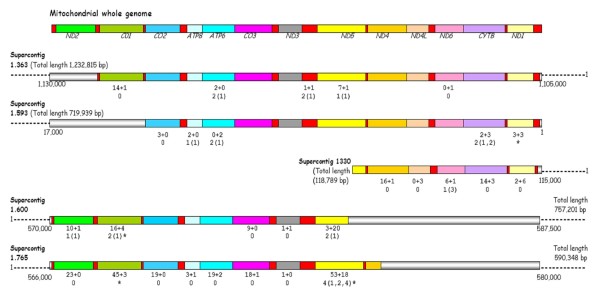
**Alignment of five supercontigs containing the longest putative Numts with the mitochondrial genome**. On the mitochondrial genome tRNA and rRNA genes are represented by red colour blocks and blocks of other colour indicate the coding genes as named. Corresponding coloured blocks on the supercontigs indicate regions of high homology with the mtDNA with mutational differences indicated below each gene as follows: Line 1 = numbers of synonymous + non-synonymous mutations, Line 2 = number of indels (length of indels in bp), * = Stop codons. Grey-shaded areas indicate regions of no homology and dotted lines represent the rest of the genomic supercontigs. The core haplotypes in clade 2 and clade 1 are identical to the corresponding *ND4 *regions on supercontigs 1.363 and 1.593, and supercontig 1.1330, respectively.

It is possible that the long Numts identified by the BLAST search may not be true Numts but instead the result of misassembly during construction of this rather large and repetitive genome sequence. To address this question we investigate the detailed composition of the five longest putative Numts with the highest matches to the mtDNA sequence, which were those mostly likely to be due to misassembly. Figure 2 shows how these putative Numts differ from the mtDNA sequence. The two longest putative Numts on supercontigs 1.593 and 1.363 are almost full length and match almost completely with the mtDNA sequence. In supercontig 1.363, the *CO1 *gene has the greatest number of mismatches with respect to the mtDNA sequence, having one coding and 14 silent mutations. The *ATP6*, *ND5 *and *ND6 *genes and the six tRNAs between *ND3 *and *ND5 *have a small number of substitution mutations but there are no stop codons. All the indels are of a single base in simple sequence repeats of the same base so it is hard to exclude sequencing error. In the case of supercontig 1.363 the mtDNA-like sequence could therefore be due to genome sequence misassembly. Supercontig 1.593 makes a more convincing case for a Numt as although there are only a small number of silent and coding mutations (in the *CO2*, *ATP8*, *ATP6*, *CYTB *and *ND1 *genes) the indels are not all single base differences in simple sequence repeats and there is a stop codon in the *ND1 *gene. The placement of the mtDNA sequence at the end of supercontig 1.1330 (Figure 2) could be a sign of misassembly. However, this sequence contains no stop codons or frameshift mutations.

Supercontigs 1.600 and 1.765 have even greater numbers of substitutions compared to the mtDNA sequence and this coupled with the presence of stop codons is very clear evidence that these are Numt sequences. In all five of these long Numts the majority of substitutions are synonymous. This is not inconsistent with their being Numts as many of the observed substitutions may have occurred in the mtDNA rather than the Numt [22,24] as, based on *Drosophila*, the mitochondrial mutation rate is likely to be ~10 times higher than the nuclear mutation rate [51]. It is interesting to note that the gene fragments at the start of the Numt on supercontig 1.363, and at the starts and ends of the Numts on supercontigs 1.600 and 1.765, have much higher numbers of substitutions (indicated in Figure 2) with respect to the mtDNA sequence than the remaining genes in these Numts. This pattern is unlikely to indicate misassembly since it is found on Numts with both high and low levels of differentiation from the mitochondrial sequence. This pattern more likely indicates a heterogenous ancestry for these Numts.

### Recovered nuclear genomic sequences

To further test the possibility of genome misassembly underlying the highly mt-DNA like sequences on Supercontigs 1.363, 1.593 and 1.1330, we designed and used primers to amplify genomic DNA fragments of 400–450 bp that span the transition between non-mtDNA-like sequence and the putative Numt (Table 2). (At the start of the Numt on supercontig 1.1330 and the end of the Numt on 1.593 there is little or no flanking regions (Figure 2) so these were not amplified.) Interestingly, primers designed to amplify across the Numt junction on supercontig 1.1330 did not recover the original sequence but instead a sequence (GenBank accession number: FJ463415) that differed from the original by 28 base substitutions and 5 indels of 1–4 bp distributed throughout the 412 bp amplified fragment. This sequence could not be found in the *Ae. aegypti *genome sequence by a BLAST search. Instead, this BLAST search identifed another similar sequence of 200 bp on supercontig 1.43 (*E *value 5 × 10^-72^) that differed from the query sequence by 12 base substitutions throughout. Rather than indicating that supercontig 1.1330 contains missassembled mtDNA sequence, the finding of similar but different junction sequences may instead indicate there are sets of related Numts. The sequences of the Numt junctions at the starts and ends of the Numt on supercontig 1.363 and the start of the Numt on 1.593 (GenBank accession numbers: FJ463412–FJ463414) fully recovered the database genome sequences. Overall, the sequence data on Numt junctions indicate that the long mtDNA-like sequences on these supercontigs are true Numts rather than the result of genome misassembly.

**Table 2:** Primers used for mtDNA specific amplification and for amplifying across Numt junctions

Region amplified	Forward primer	Reverse primer	Fragment size (bp)
Clade 2 specific *ND4 *gene region	ATGATAATTATACAATGAATTTTA	AACTCCCCCAATTAAGCTAATACTA	282
Start junction of Numt on 1.363	GCTGGTGTGTGCATGAAACTAATC	TCGCGATTAAATGGCTGAAG	406
End junction of Numt on 1.363	TCAAACGGACGAAGTTTGAGACAG	AAACCATGCCATTCCTTGAG	444
Start junction of Numt on 1.593	ATAAGTGAGCCGAAGACACCGAG	GTCCGGTCTAGGGTCCTTTC	413
Start junction of Numt on 1.1330	TCACAATCACAGCCACTTTTCC	CCTATTCAAAACAGGTTTCGTTCAAG	412

### Deep clade structure of putative mitochondrial haplotypes

**Figure 3:**
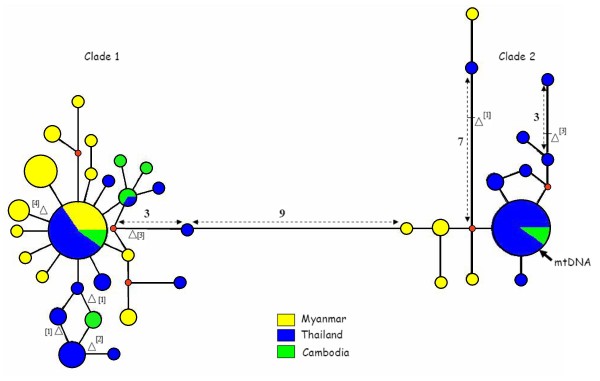
**Median-joining (MJ) network for *ND4 *putative mtDNA sequences from 141 individuals of *Ae. aegypti *from Southeast Asia**. Note: Each circle represents a unique haplotype. Circle size is propotional to haplotype frequency and circle colour indicates the country of origin. The lengths of branches between haplotypes are proportional to the number of mutations. Numbers along branches indicate 3 or more mutational differences between the haplotypes. Δ refers to non-synonymous mutations with amino acid changes: [1]  Valine to Isoleucine; [2] Proline to Serine; [3] Isoleucine to Valine; [4] Serine to Proline. As indicated by an arrow, the published mtDNA sequence corresponds to the clade 2 core sequence.

To determine if Numts may be a problem in mtDNA-based population genetic studies of *Ae. aegypti*, we amplified and sequenced the putative mitochondrial gene nicotinamide adenine dinucleotide dehydrogenase (NADH) subunit 4 (*ND4*) from natural populations of *Ae. aegypti*. A total of 763 usable bases of the *ND4 *gene was generated from 166 *Ae. aegypti *mosquito individuals collected from Myanmar, Thailand and Cambodia. Of these, 141 sequences were unambiguous and corresponded to 38 unique haplotypes (GenBank accession numbers: FJ428759–FJ428796) varying in frequency from 1 to 50. In a median-joining (MJ) network, these haplotypes fell into two divergent clades with both clades containing individuals from all three countries (Figure 3). The core sequences in each clade were separated by 17 mutations, 16 of which were synonymous changes. The one non-synonymous mutation was a conservative amino acid change from Ile to Val. The A+T content of the clade 1 and clade 2 core haplotypes was 76.4% and 75.6% respectively, typical of insect mtDNA. There were no frameshift or stop codon mutations within or between the two clades. Within clade 1 there were four non-synonymous mutations and within clade 2, two non-synonymous mutations. Clade 1 and clade 2 have somewhat similar topologies with a high frequency core haplotype. However, clade 1 has a more star-like structure with many individuals differing from the core by 1–4 mutations whereas in clade 2 several haplotypes are quite divergent from the core.

In addition to the 141 clear sequences we also obtained 25 ambiguous sequences, corresponding to ~15% of the total sample. The ambiguous sequences possessed double peaks at 9–17 sites all of which were sites that differentiated the two clades. Since the forward primer used here differed from those used in previous studies [41] we repeated the amplification and sequencing of 12 individuals with ambiguous sequences using the orginal primers that amplified a shorter 359 bp fragment corresponding to the 3' end of our amplicon. Even for these shorter fragments, the ambiguities were consistently present, in the same individuals and involving the same sites that differed between the two clades. This situation was not changed even by redesign of the reverse primers. These sequence ambiguities indicate that Numts are being amplified instead of, or together with, *Ae. aegypti *mtDNA. The core sequence of clade 2 has a 100% sequence identity with the mtDNA sequence of *Ae. aegypti *(GenBank accession number: EU352212) which indicates that clade 1 most likely comprises Numts.

### Confirmation that clade 1 contains Numt sequences

**Figure 4:**
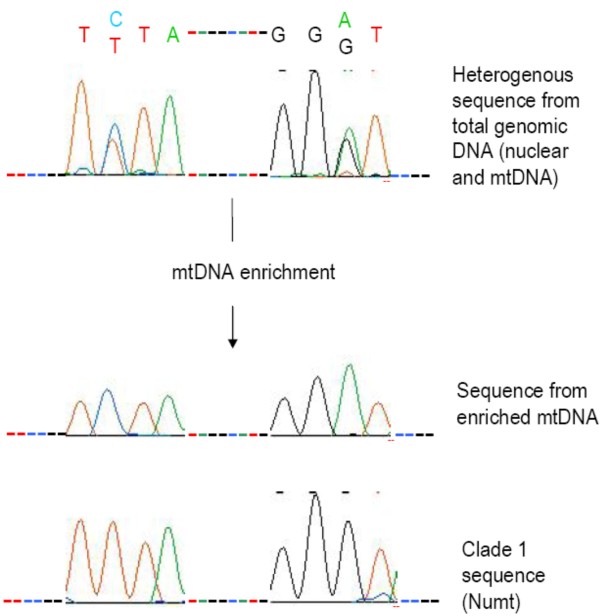
**Example of the removal of a clade 1 Numt sequence from a heterogenous sequence by mitochondrial enrichment**. The electropherogram profile shows superimposed peaks at positions 159 and 194 of *ND4 *heterogenous sequences. The same pattern is found in the complementary strand (data not shown).

Mitochondrial DNA was enriched from five fresh individuals from Chiang Mai, Thailand. The *ND4 *gene region was amplified and sequenced from both the total genomic DNA (prior to purification) and the enriched mtDNA for all individuals. The core clade 2 sequence was obtained for three individuals for both the total genomic DNA and the enriched mtDNA. However, the total genomic DNA of the remaining two individuals yielded ambiguous sequences that were composites of a clade 1 sequence and the clade 2 core sequence. Following enrichment, the mtDNA fraction generated a clear clade 2 sequence (Figure 4). In addition to confirming the sequence of the mtDNA, this demonstrates that the clade 1 sequences in these two individuals are due to Numts.

If all the clade 1 sequences obtained from the Southeast Asian mosquitoes were Numts we would also expect an mtDNA sequence to be present in each individual. We therefore developed primers that amplify 282 bp of the *ND4 *gene from clade 2 (i.e. known mtDNA-like) sequences only (Table 2). These clade 2/mtDNA-specific primers had 3' termini at bases that distinguished clade 2 from clade 1. The PCR conditions under which these primers were specific for the clade 2/mtDNA sequence were determined using clade 1 and clade 2 PCR products as templates. Under these conditions the mtDNA-specific primers generated PCR products from all individuals that had previously given rise to unambiguous clade 1 sequences using the original primers. Nine of these amplicons were sequenced and all sequences were confirmed to fall into clade 2. For sequences of both clades to be present within an individual, at least one of the sequences must be a Numt. Based on the results of the mtDNA enrichment which showed clade 1 Numts and that the known mtDNA sequence corresponds to clade 2, the most parsimonious explanation is that all the clade 1 sequences are Numts. However, we cannot exclude the possibility that the clade 2/mtDNA specific primers are amplifying clade 2-like Numts such as those detected on supercontigs 1.593 and 1.363 by the BLAST searches.

### Sequences from cloned PCR products

**Figure 5:**
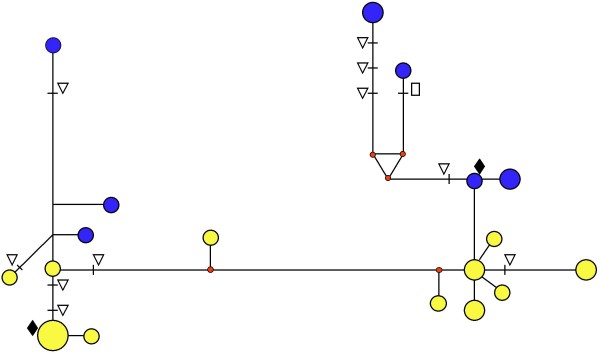
**Median-joining network showing mutational differences among clones of *ND4 *sequences from two mosquito individuals**. Haplotype colour indicates which of the two individuals a sequence originated from. Symbols denote the following: € stop codon; ∇ amino acid change; ◆ core clade 1 and core clade 2 haplotypes from Figure 3.

To further assess the extent of Numt presence in *Ae. aegypti *we also cloned and sequenced PCR products of the *ND4 *gene from two individuals from which we had obtained ambiguous sequences. We expected Numts to be characterised by being present as multiple, not necessarily closely related, sequences within an individual and with the possible presence of stop codon and/or frameshift mutations. Mitochondrial genes on the other hand should be comprised primarily of one sequence with the possible existence of sequence variants differing by one (or at most two) mutations due to *Taq *error during amplification captured by the cloning process, assuming an error rate of 7.3 × 10^-5 ^per bp per duplication [52]. This logic was also used by Arctander [24] to distinguish a Numt and mitochondrial sequence. However, contrary to our expectation, we found that for both individuals each clade contained multiple, divergent cloned sequences with around one third of the mutations being amino acid changes (Figure 5). Those sequences which cluster within a few mutations of the sequence inferred by direct sequencing of the corresponding original PCR product could just be the result of *Taq *error. However, even if the *Taq *error rate was higher than predicted, there are at least four outlier sequences that differ by 7–9 mutations from the original PCR-based sequence which cannot be attributed to *Taq *error alone. For example, in Clade 2 one haplotype differs from the putative mtDNA sequence in that individual by seven mutations including one non-synonymous substitution and one stop codon. These divergent sequences are most likely Numt sequences, indicating that there are both recently derived clade 1- and clade 2-like Numts. These novel variants of the *ND4 *gene could not be found in the genome sequence by BLAST searches. This may simply be because the Southeast Asian individuals we sequenced differ in genomic composition from the sequenced strain or they may be revealed in the future when a more complete assembled version of the genome is released.

## Discussion

### High numbers of Numts in the Ae. aegypti nuclear genome

The results of the BLAST searches demonstrate the presence of high numbers of Numts in the genome of *Ae. aegypti *with Numts detected on a total of 146 different supercontigs. This is comparable to other insects that are considered to have large numbers of Numts including the honeybee, *Apis mellifera*, and the *Tribolium *flour beetle which have Numts at an estimated 575 and 57 genomic locations, respectively [30]. It is notable that other dipterans studied to date, *D. melanogaster *and *An. gambiae*, which have few or no Numts, have much smaller genomes than *Ae. aegypti*; 176 Mb and 278 Mb compared to 1.3 Gb for *Ae. aegypti*. Almost half the genome of *Ae. aegypti *is composed of transposable elements [34] which may indicate that this species is generally unable to rid itself of non functional DNA, including Numts.

In agreement with studies in other taxa [e.g. [30]], the majority of the Numts detected here were short. However, we also noted many long Numts with six being greater than 7 kb in length with the two longest on supercontigs 1.363 and 1.593 (Figure 1). Although these latter two differed very little from the mitochondrial sequence they appear to be real rather than the result of misassembly as there are some frameshift and stop mutations (albeit very few in number) and the presence of Numt-genome junction points was confirmed by amplification and sequencing. At 15.46 kb and 13.07 kb, the Numts on supercontigs 1.363 and 1.593, respectively, are amongst the longest reported to date. Other species with long Numts are humans [36], *Arabidopsis*, rice, house mice [32], domestic cats [19], and voles [53] that have Numts of 14.65 kb, 20.13 kb, 13.32 kb, 12.4 kb, 7.9 kb and 4 kb in length, respectively.

In general, as the Numts in *Ae. aegypti *get progressively shorter in length their similarity to the mitochondrial sequence decreases (Figure 2). This is consistent with the repeated movement of long mtDNA sequences to the nucleus which then decay through time becoming both fragmented and mutated. This is also consistent with our observation that, despite some variation among genes, all the mitochondrial genes are represented in Numts in our study. This is similar to the situation in humans [36,54] and mouse and *Ciona *[32] but contrasts with several other studies which have noted a very marked preference for particular mtDNA genes to become Numts [29,30]. The situation in which the Numt on supercontig 1.1330 was found to have a homologous Numt with the same end point but slightly different genomic flanking sequence has been found previously [54]. Such Numt copies could have arisen from the secondary integration of Numts from one nuclear genomic location to another [28,36,55]. Genomic rearrangements involving Numts seem common [36]. In accord with this, the pattern seen here of high substitution rates at the extreme ends of three of the long Numts indicates that these Numts could have been formed by the homologous recombination of long mtDNA sequences into genome positions that already contained Numts. However, it is also difficult to exclude the possibility of genome misassembly in giving rise to this pattern. Overall, our data lead to the conclusion that Numt evolution in *Ae. aegypti *is a very complex and dynamic process.

### Origin of Clade 1 and Clade 2 type Numts

The genome sequence was obtained from a highly inbred strain of *Ae. aegypti *[34]. It seems unfeasible that this strain could have maintained two mtDNA sequences, corresponding to clades 1 and 2, through successive bottlenecked generations. The evidence presented here demonstrates clearly that at least some, and possibly all, clade 1 sequences originate from Numts and not from mtDNA. Not only do the clade 1 sequences generated from *ND4 *PCR products (Figure 3) correspond to the sequences removed by mtDNA enrichment and the Numt on supercontig 1.1330, but clade 2 sequences were also found in all individuals that generated clade 1 sequences. The clade 2 sequences generated from *ND4 *PCR products (Figure 3) correspond to the reported mtDNA sequence. However, it is possible that not all of these clade 2 sequences have arisen from mtDNA as the data indicate that there are also very recently derived Numts: firstly, the putative Numts on supercontigs 1.363 and 1.593 are almost identical to the mtDNA sequences; and secondly, the clade 2 sequences of cloned PCR products have high numbers of mutations (Figure 5). Although it could be argued that these findings are the result of misassembly of the nuclear genome sequence and high *Taq *error rate, respectively, these do not appear to be the most parsimonious arguments.

The sites that differ between our clade 1 and clade 2 sequences are the same as those found in a large number of other studies [37-41,56] using the putative *ND4 *mtDNA gene. In this and previous studies, the primers used were designed using mtDNA sequences from related taxa (*Ae. albopictus*). It has been noted that this strategy is inherently prone to the co-amplifcation of Numts [57]. Many of the previous studies e.g. [37-39,41] relied on single-strand conformation polymorphism (SSCP) coupled with sequencing of a few individuals. If co-amplifying Numt sequences were present and formed additional bands on the gene they may have been disregarded, as SSCP gels can contain background bands due to the same sequence adopting alternative conformations. This may explain why, besides this one, there has only been one other report [47] of heterogenous sequences using this marker. In this latter case the authors attributed the heterogenous sequences to heteroplasmy because the mixed sequences contained the variation found in their clade 1 and clade 2 sequences which they believed to all be true mtDNA.

There are at least two possible explanations for the origin of the clade 1 and clade 2 type Numts. Firstly, the Numts could have originated from the same species at different time points with the clade 1 sequences being entirely due to Numts and transferring at an earlier time. The deep clade structure would therefore have been formed by the movement of clade-1 like mtDNA into the nuclear genome, presumably in Africa of the order of one million years ago (making the assumption that divergence rates will have been similar to those of mtDNA estimated at 2.3% per million years [58]). This might at first appear inconsistent with the lack of amplification of other recently derived Numts, particularly of sequences intermediate between clades 1 and 2 that might be expected from continual mtDNA transfers to the nucleus. However, the ease with which clade 1 Numts are amplified could be explained by their being present in multiple copies in the genome, for example, due to secondary integration as suggested above. The one coding and 16 silent substitutions separating clade 1 and clade 2 sequences indicate the action of purifying selection. These substitutions would therefore have accumulated primarily along the clade 2 mtDNA lineage. This is consistent with the clade 1 sequences being multi-copy Numts as their independently acquired substitutions would not be apparent in sequenced PCR products. The star-like structure of the clade 1 sequences in the haplotype network is, however, hard to explain under this scenario.

A second possible explanation is that both clade 1 and clade 2 Numts are of recent origin but have originated from different source populations/species with divergent mtDNAs. A likely (but not only) candidate for a source of divergent mtDNA is the forest form of *Ae. aegypti *from Africa. The domestic form of *Ae. aegypti *studied here derives from Africa where there is known to be ongoing gene flow between it and the forest form [43]. Putative mtDNA *ND4 *haplotypes of the forest form are shared with those of the domestic form in West Africa [59] and (from a comparison with our sequences) these comprise both clade 1 and clade 2 sequences. However, it is possible that the forest and domestic form have recently made secondary contact following a period of allopatry during which their mtDNA could have diverged. Clade 1 and clade 2 Numts could have arisen pre or post secondary contact and in either the forest and/or the domestic form.

These alternative hypotheses for the origin of the clade 1 and clade 2 Numts (single source at different times versus different sources and recent origin) could be distinguished by sequencing enriched mtDNA from a large number of individuals, ideally including those of the forest form. Under the second hypothesis at least some individuals should have clade 1 type mtDNA. This should be performed in conjunction with amplifications of the total genomic DNA using clade specific primers to determine if clade 1 or clade 2 Numt sequences are present.

### Implications for inference of population history and genetic structure

The unknowing inclusion of Numts sequences in mtDNA based analyses could lead to mistaken inferences of population structure and population history. Since *Ae. aegypti *mosquitoes have spread from Africa to the rest of the Tropics relatively recently, the deep clade structure found in previous studies has often been interpreted to suggest colonization from at least two different source populations (and/or by many individuals) [38,40,46,60,61]. However, the deep clade structure could instead be due to the presence of Numts. In this case, the two deep clades could also mistakenly be interpreted to indicate the presence of two distinct species outside of Africa. This exemplifies the problem pointed out by Song *et al*. [62] that the use of mtDNA genes for DNA barcoding could lead to the overestimation of species numbers.

Although the putative mtDNA sequences can clearly detect genetic population structure among regional populations of *Ae. aegypti *(Table 3), the reliability of differentiation estimates would be affected by the inclusion of Numts. This is particularly the case if the proportion of Numt amplification is not consistent across populations. This is suggested here by the notably lower proportion of clade 1 amplification from the northwestern Thai population (χ^2 ^= 22.02, df = 3, *P *< 0.001). If the clade 1 sequences are actually Numts, this results in a dramatic increase of the apparent level of differentiation between the northwest and eastern Thai populations (from *F*ST of 0.088 to 0.28; Table 3).

**Table 3:** Population pairwise *F*ST values estimated using clade 1 and clade 2 sequences

Sample locations	Sequences used	Myanmar (Yangon)	northwest Thailand (Chiang Mai)	northeast Thailand (Ubon Ratchathani)
northwest Thailand (Chiang Mai)	Clade 2 (mtDNA-like)	0.323*		
	Clade 1	0.248*		
northeast Thailand (Ubon Ratchathani)	Clade 2 (mtDNA-like)	0.082	0.088	
	Clade 1	0.043	0.282*	
Cambodia (Battambang)	Clade 2 (mtDNA-like)	0.021	0.110	-0.105
	Clade 1	0.136	0.152*	0.136*

* Significant (*P *< 0.01)

There are a number of means by which Numt co-amplification can be limited such as mitochondrial purification before DNA extraction, long PCR, reverse transcriptase PCR, or using tissue rich in mtDNA (e.g. muscle) but none are guaranteed to absolutely overcome the problem ([22] and references therein). Perhaps the simplest solution to the problem providing that the Numts are monophyletic with respect to the mtDNA sequences, is their removal by restriction enzyme digestion or their avoidance by the use of mtDNA-specific primers [22]. However, these possible solutions will not work here if, as the BLAST results and sequences of clones indicate, there are very recently derived Numt sequences. We therefore advocate that future studies of population structure in *Ae. aegypti *use other markers. With the genome sequence available there are a large number of nuclear markers to choose from.

## Conclusion

Using a variety of approaches we conclude that Numts are prevalent in *Ae. aegypti *and that Numt evolution in *Ae. aegypti *is a very complex and dynamic process. These data also show that the deep clade structure we and others have been obtaining when performing supposedly mtDNA-based studies in *Ae. aegypti *may in fact be due to Numts. Some interpretations of the population and colonisation history of *Ae. aegypti *made to date may therefore be unreliable. We advocate that future studies of genetic population structure and gene flow in *Ae. aegypti *avoid using mtDNA data and use nuclear markers instead.

## Methods

### BLASTN searches of Ae. aegypti genome sequence

This study utilized the genomic sequence of *Ae. aegypti *(version-AaegL1) sequenced from the Liverpool LVP strain to approximately 8× coverage by the Broad Institute and The Institute for Genomic Research (TIGR). The genome sequence has over 1.31 billion bp and comprises 4,758 supercontigs but these have not yet been physically mapped to chromosomes .

This genomic sequence was searched for Numts by BLASTN [63] on VectorBase for each of the 13 protein-coding genes, 22 tRNA genes and 2 rRNA genes from the *Ae. aegypti *mitochondrial genome sequence of 16,655 bp (GenBank accession number: EU352212). Threshold levels for the inference of Numts from BLASTN hits were taken as expectation values (*E *values) of 10^-4 ^or 10^-14^, as used in other studies [30,32,64]. We determined the extent to which the hits against single genes were contiguous and due to single large Numts by carrying out alignments of the mtDNA genome with the regions of supercontigs containing multiple hits using ClustalX (1.83) [65,66]. The aligned sequences were visualized in MEGA ver. 4 [67] to determine the lengths of Numt sequences and to identify the Numt-supercontig junction points. The same software was also used for the translation of protein coding sequences to identify missense mutations and stop codons.

### Extraction of genomic DNA, PCR amplication and direct sequencing

DNA was extracted from individual mosquitoes using a standard phenol/chloroform method [68]. The final DNA pellet was suspended in 20 μl water (dH_2_O) and was diluted 1:20 to make a working solution. A fragment of the nicotinamide adenine dinucleotide dehydrogenase (NADH) subunit 4 mitochondrial (*ND4*) gene was amplified using the primers: ND4F (5'-GTTTAGATATARTTTCTTAYGG-3') and ND4R (5'-CTTCGDCTTCCWADWCGTTC-3'). The ND4R primer was the same as that used in previous studies in Mexico [37,49], Thailand [38] and Venezuela [41]. The ND4F primer used here was designed in Primer3 ver. 0.4.0 [69] from an alignment of the *ND4 *region of the mtDNA of *Aedes albopictus *(GenBank accession number: AY072044), *Anopheles gambiae *(GenBank accession number: L20934) and *Anopheles quadrimaculatus *(GenBank accession number: L04272). The combination of primers used here therefore amplified a fragment that had the same 3' end as that of the previous studies but which was longer, being 763 bp rather than 359 bp in length.

The concentrations of the PCR reactants were 1 × ammonium buffer (CLP, Northampton, UK), 2.5 mM MgCl_2_, 200 μM dNTP and 0.4 μM primers. Amplifications were carried out in 50 μl volumes using 1 μl of template DNA. The PCR programme was modified from the previous Thailand and Mexico studies [37,38,49]. In an intial hot start, the reagents were heated to 95°C for 2 mins prior to the addition of 1.25 units of Thermoprime plus DNA polymerase (CLP, Northampton, UK). This was followed by 38 amplification cycles consisting of 30 sec at 92°C, 1 min at 50°C and 40 sec at 72°C, followed by a final extension for 5 mins at 72°C. PCR reactions were carried out on a GeneAmp^® ^PCR System 9700 thermocycler (Applied Biosystems, Warrington, UK). PCR products were purified using Montage columns (Millipore, Billerica, MA, USA) and sequenced in both directions (Macrogen Inc., Seoul, Korea). DNA sequences were assembled using the Sequencher multiple sequence editor program ver. 4.5 (Gene Codes Corporation, Ann Arbor, USA) and checked manually. A total length of 763 bp of *ND4 *sequence was obtained from each individual.

### Genetic analyses

Median joining networks were constructed using DNA Alignment ver. 1.0.0.3 and Network ver. 4.1.0.8 [70]. Genetic differentiation between pairs of populations (*F*_ST _values) was estimated from the sequence data using analysis of molecular variance (AMOVA) in ARLEQUIN version 3.01 [71]. Significance was estimated by 1000 permuations of the haplotypes between the populations.

### Cloning and sequencing of PCR products

PCR products of the *ND4 *gene from individuals that yielded a mixed clade 1 and clade 2 sequence were cloned using the pGEM-T Vector System II kit according to the manufacturer's instruction (Promega Corporation, Madison, WI, USA). The same PCR primers were used to amplify the cloned *ND4 *gene fragments from colonies containing inserts. Colony material was prepared for PCR by heating a small portion of an individual colony in 20 μl dH_2_O to 95°C for 5 mins and 0.5 μl of this was used in PCR reaction volumes of 25 μl. The amplifications were performed using the same reactant concentrations as above. The PCR conditions were initial denaturation at 94°C for 5 mins followed by 30 cycles of 92°C for 30 sec, 52°C for 1 min and 72°C for 40 sec, with a final extension at 72°C for 7 mins. The PCR products were purified and sequenced and the same procedures as outlined above were used to check sequences and to construct a median joining network.

### Fresh mtDNA separation and PCR amplification

Laboratory-reared freshly killed *Ae. aegypti *mosquitoes originating from Thailand were used for the isolation and extraction of enriched mtDNA using an alkaline lysis method [72]. For each individual, DNA was extracted using a standard phenol/chloroform DNA extraction method [68] from both the starting homogenate (which contained both nuclear and mtDNA) and from the final mtDNA-rich supernatant. PCR amplification and sequencing of the *ND4 *region was carried out as above.

### Additional PCR reactions

Primers designed and used for the specific amplification of clade 2 *ND4 *gene sequences and for the amplification of Numt junctions are given in Table 2 (see main text for details of their application). PCR was performed as before with the exception that no hot start was used and that the annealing temperature was increased to 55°C to achieve high specificity.

## Authors' contributions

TH carried out the molecular genetic studies, data analysis, some of the fieldwork and drafted the manuscript. CW conceived the study, led its design and co-ordination and helped to draft the manuscript. WTL, PS, DS and MSC participated in study design and co-ordination. TS, SM, and PS conducted fieldwork to collect mosquito specimens. All authors read and approved the final manuscript.
